# Chikungunya virus with E1-A226V mutation causing two outbreaks in 2010, Guangdong, China

**DOI:** 10.1186/1743-422X-10-174

**Published:** 2013-06-02

**Authors:** De Wu, Yonghui Zhang, Qiong ZhouHui, Jing Kou, Wenjia Liang, Huan Zhang, Corina Monagin, Qiaoli Zhang, Wenjie Li, Haojie Zhong, Jianfeng He, Hui Li, Songwu Cai, Changwen Ke, Jinyan Lin

**Affiliations:** 1 The Center for Disease Control and Prevention of Guangdong Province, 160 Quxian Road, Dashi Street, Panyu District, Guagnzhou, Guangdong, 511430, China; 2 Metabiota, San Francisco, CA, 94104, USA; 3 The Center for Disease Control and Prevention of Dongguan, Guangdong, China; 4 The Center for Disease Control and Prevention of Yangjiang, Guangdong, 529500, China

**Keywords:** Chikungunya virus, Chikungunya fever, Phylogenetic analysis, Molecular epidemiology

## Abstract

**Background:**

CHIKV is a mosquito-borne emerging pathogen that has a major health impact in humans in tropical zones around the globe. A new variant of the virus, E1-A226V caused a large outbreak in the Indian Ocean islands and India from 2004–2007. CHIKV outbreak was initially reported in Dongguan region of Guangdong in 2010 in China, another smaller CHIKV outbreak was found in Yangjiang region of Guangdong two weeks later. The viral agent causing the two outbreaks was inferred to be the new E1-A226V variant and Yangjiang CHIKV might be introduced from Dongguan. To confirm the hypothesis and determine the origin of CHIKV causing the outbreaks, we described Yangjiang outbreak in this study, and the molecular characterization of CHIKV from Yangjiang and Dongguang outbreaks were analyzed.

**Results:**

27 clinical cases of CHIK fever were reported in outbreak in Yangjiang region. Sera sample from 12 clinical cases were collected from the outbreak, and nucleic acid and antibody tests for CHIKV were performed using Real-time RT-PCR and indirect immunofluorescence. Positive samples of Real-time RT-PCR were subjected to viral isolation. The results showed 3/12 samples positive for Real-time RT-PCR. 7/12 and 4/12 samples were positive for IgM and IgG against CHIKV respectively, two virus strains were isolated. Four viral genomes from Dongguan and Yangjiang were sequenced, characterized and phylogeneticly analyzed. Phylogenetic analysis revealed that the four seqeunced viruses had the closest relationship (99.4~99.6% identify) with the Singapore 2008 isolate belonging to the Indian ocean clade. A common mutation at the site of the E1-A226V was observed among four viruses. Four and three aa substitutions were detected in the CHIKV sequence from the Dongguan and Yangjiang outbreak strains respectively.

**Conclusion:**

CHIKV with an E1-A226V mutation that originated from Southeast Asia isolates caused two outbreaks in China in 2010, and originated from two different infectious sources.

## Background

Chikungunya virus is an insect-borne virus, of the genus Alphavirus, that is transmitted to humans by virus-carrying *Aedes* mosquitoes [1]. Human infections caused by Chikungunya virus were reported for the first time in East Africa in 1952–53 during an epidemic of fever that developed along the border between Tanzania and Mozambique [2]. Retrospective case reviews have suggested that CHIKV epidemics occurred as early as 1779 but were frequently documented inaccurately as dengue outbreaks [3]. Between the 1960s and 1990s, the virus was isolated repeatedly from numerous countries in Central, Southern and Western Africa [4]. In Southeast Asia, the first outbreak was reported in Bangkok in 1958 [5] followed by frequent outbreaks in India [6], Indonesia [7], Myanmar [8], Malaysia [9], Singapore [10], Thailand [11], Cambodia [12] and Vietnam [13]. However, no outbreak due to the local transmission of CHIKV was reported in China before 2010.

Mutations in CHIKV, climate change, increasing globalization, and increasing ease of travel have favored the continuing spread of mosquitoes to non-indigenous habitats [4,14]. The new E1-A226V variant enhanced the replication and dissemination of CHIKV in *Ae*.*albopictus*[15] and caused the largest outbreak in the Indian Ocean islands and India during 2005–2007 [16,17]. This new virus lineage was introduced into India, Thailand, Malaysia and Réunion Island during 2008 and 2010 [18-21]. With an increase in global travel, the risk that CHIKV will continue to spread to non-endemic regions, such as China, has also increased [22]. Two imported CHIKV cases from Singapore and Indonesia in 2006 and 2007 were reported in Taiwan, China [23], and the importation of five chikungunya fever cases from Sri Lanka and Malaysia to mainland China during March, October, and December of 2008, were reported [24].

CHIKV outbreak was initially reported in the Wanjiang community of Dongguan city in early October, 2010, in Guangdong, China [25], coincidentally, another small outbreak was confirmed at the Huahong community of Yangjiang city tow weeks later. China is a non-indigenous region of CHIKV and two key questions emerged regarding the origin and adaptability of the virus that initiated the two outbreaks. In previous studies, genetic and mutation analysis were used to study the origin and mutation of the virus [14,26]. In order to further clarify the two questions in our study, we sequenced the complete genome of four isolates obtained from the Dongguan and Yangjiang outbreaks, and compared these sequences with worldwide strains from GenBank to infer the origin of the virus and whether the viral changes had occurred during the course of the outbreaks.

## Results

### Two outbreaks

**Figure 1:**
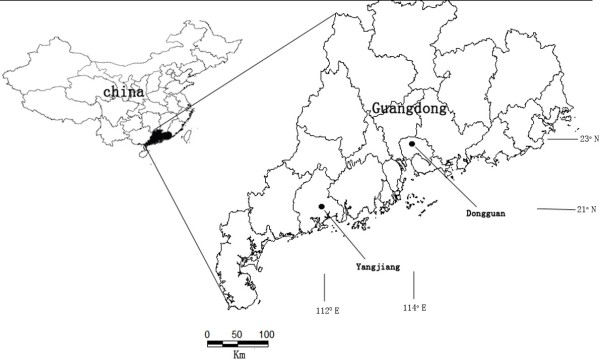
Locations of outbreaks of chikungunya fever in Dongguan and Yangjiang, Guangdong, China, October 2010.

**Figure 2:**
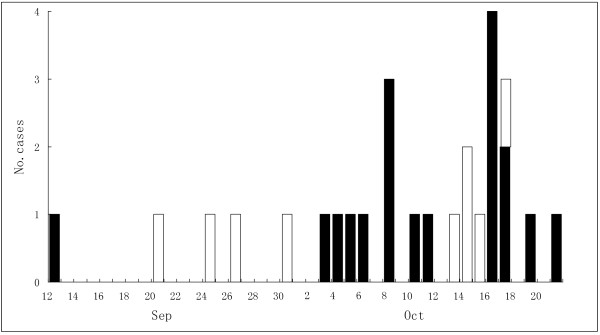
**Number of reported cases of CHIK fever between September and October, 2010 in Yangjiang.** Black bar sections indicate clinical cases and white bar sections cases confirmed by laboratory detection.

A bigger CHIKV outbreak occurring in Dongguan city was described in previous study [25]. A smaller CHIKV outbreak was confirmed in a construction site in the Huahong community in Yangjiang city on October 18th, 2010. 227 people resided or worked within the construction site at the time. Yangjiang city is approximately 280 kilometers from Dongguan city where the first CHIKV outbreak occurred in, located in the western region of Guangdong Province [25] (Figure 1). Retrospective investigation showed the first definitive case of Chikungunya fever on September 12th. Cases increased after 23 September, and peaked during the period of October 9th and 17th. The last case occurred on October 21st (Figure 2), with a total of 27 cases (17 male and 10 female) reported during the outbreak. The attack rate was calculated at 11.9%. Most patients recovered within one week after the onset of symptoms. No fatal cases were reported in the outbreak.

### Vector monitoring

To monitor density of *Aedes* mosquitoes and evaluate the risk of dengue-virus transmission in Guangdong, the Breteau index (BI) has been used as a mosquito density investigation tool for many years. BI is defined as number of positive containers for *Aedes* per 100 houses. BI was investigated for one month after the two CHIKV outbreaks were confirmed in two communities as well as their neighbor communities. No *Ae*. *aegypti* were found during monitoring, and *Ae*. *albopictus* was found to be the predominant species. An average BI of 126 was observed in the Huahong communities before control measures were implemented. The BI began to notably decrease after control measures were implemented. Average BI after control measures was implemented were found to be 2.1 in the Huahong communities.

### Sample collection and IgM and IgG detection

A total of 12 sera samples were collected from patients between the ages of 32–70 years with dengue-like symptoms in the outbreak. The patients were comprised of 8 males and 4 females. Five of 12 samples were collected during the acute phase (1–9 days after onset of symptoms) and 7 of the 12 samples were collected during the convalescent phase (10–28 days after onset of symptoms). For serologic diagnosis, these 12 samples were subjected to an indirect immunofluorescence test (IIFT) and an enzyme linked immunosorbent assay (ELISA) for CHIKV and Dengue virus IgM and IgG antibody respectively. The results proved that 7 and 4 samples were positive for CHIKV IgM and IgG respectively. All of the sera were negative for dengue IgM and IgG antibodies.

### Real-time RT-PCR and virus isolation

In order to diagnose the suspected cases at the nucleic acid level, improve the CHIKV isolation rate, and reduce labor intention, 12 samples collected from the Yangjiang outbreaks were subjected to Real-time RT-PCR (Table 1). The results showed that 3 samples collected from cases in the acute phase were positive. Three Real-time RT-PCR positive specimens were inoculated on C6/36 and BHK-21 cell lines to isolate CHIKV. Two CPEs were observed on both C6/36 and BHK-21 cells after 4–7 days incubation. RNA was extracted from the supernatant with CPE and analyzed by Real-time RT-PCR and nucleotide sequencing. CHIKV was confirmed in these CPE samples.

**Table 1:** **Characteristics of CHIKV cases and serum sample detection for CHIKV in Yangjiang of Guangdong**, **China**, **2010**

**Cases No.**	**Sex/age,y**	**Symptom onset**	**Sampling date**	**Sign and syndrome**						**Test**	
				**Fever**	**RF**	**HC**	**Arth**	**Myal**	**MR**	**Real-time RT-PCR/ VI**	**IgM/IgG**
D10131	M/38	12 Oct	18 Oct	+	+	-	+	+	-	-/ ND	-/-
D10132	M/49	14 Oct	18 Oct	+	+	-	+	+	-	-/ ND	+/+
D10133	M/46	16 Oct	18 Oct	+	-	+	+	-	-	-/ ND	-/-
D10134	F/42	17 Oct	18 Oct	+	-	-	+	-	+	+/+	-/-
D10135	M/32	8 Oct	18 Oct	+	-	-	-	+	-	-/ ND	-/-
D10136	F/44	26 Sep	18 Oct	+	+	-	+	+	-	-/ ND	+/+
D10137	M/48	20 Sep	18 Oct	+	-	-	+	-	+	-/ ND	+/+
D10139	M/41	15 Oct	19 Oct	+	-	-	+	-	+	+/+	-/-
D10140	F/35	13 Oct	19 Oct	+	-	+	+	-	-	-/ ND	+/-
D10141	M/38	24 Sep	19 Oct	+	-	-	+	-	-	-/ ND	+/+
D10142	F/47	30 Sep	19 Oct	+	+	-	+	+	+	-/ ND	+/-
D10143	M/70	14 Oct	19 Oct	-	-	-	+	-	-	+/-	+/-

Abbreviation*: F* female; *M* male; *IgM* Immunoglobulin M; *IgG* Immunoglobulin G; “-”, Negtive; “+”, Positive; *ND* not done; *RF* Red face; *Arth* Arthralgia; *Myal* Myalgia; *HC* Headache; *MR* Maculopapular rash; *VI* virus isolation.

### Genome amplification and phylogenetic analysis of CHIKV

Four complete genome sequences of CHIKV, which were isolated from two outbreaks in Dongguan and Yangjiang, were obtained from the overlapping amplicons using 14 primer sets total. Their genome sizes had lengths of 11704~11720 bp with a short 25~54 nt at the 5′-UTR, and 456~489 nt at the 3′-UTR. The structural polyprotein and nonstructural polyprotein were encoded by two long open reading frames of 3747 nt and 7425 nt, corresponding to 1249 aa and 2475 aa respectively.

**Figure 3:**
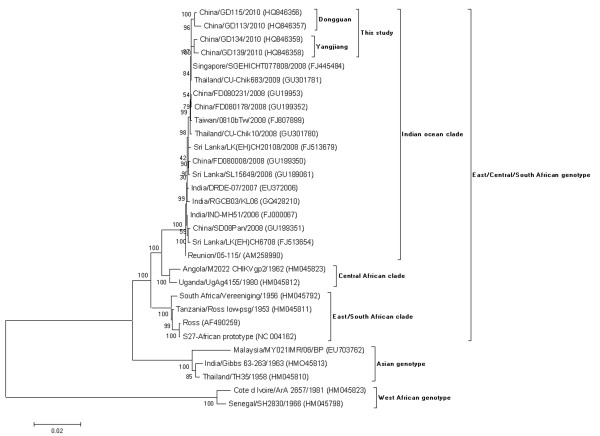
**Phylogenetic analysis based on the complete genome (11,700 nucleotides).** The nucleotide sequences were analyzed using a MEGA 4.0 package. The phylogenetic tree was constructed by Neighbor-joining cluster analysis with the bootstrap option. The numbers at the branching points are bootstrap values estimated with 1,000 pseudo-replicate data. The last numbers of each strain name indicate the year of isolation. The locations are shown to the left of the strain name. GenBank Accession numbers are given in parentheses.

Phylogenetic analysis was performed using the complete genome of 4 strains from this study and 26 worldwide strains from different CHIKV outbreak events and regions in GenBank. The analysis showed 30 sequences were divided into three genotypes: the West African (WAf) genotype, East/Central/South African (ECSA) genotype, and Asian genotypes. ECSA was the most complex genotype, consisting of three clades: Central African clade, East/South African clade, and the Indian Ocean clade, consisting of more diverse strains and including all CHIKV isolates from 2005–2010. From the phylogenetic analysis, the Dongguan and Yangjiang 2010 isolates were grouped in the Indian Ocean clade, including CHIKV isolates from China, Singapore, Thailand, Sri Lanka, Reunion, India and Taiwan isolated since the year 2005. These strains have the closest relationship with the Singapore isolate (FJ445484) (Figure 3). The genome sequence identity values among 30 strains were 94.1-100% (data not shown). Two genomes from Dongguan had a range of 99.2-99.4% identity values with two Yangjiang genomes, and 99%-99.4% identity values with four imported genomes from 2008 in China. A higher range identity of 99.4%-99.6% was observed between four 2010 outbreak isolates and the Singapore isolate (FJ445484).

Four and three common amino acid residue changes were observed in polyproteins within the two isolates from Dongguan and Yangjiang compared to the Singapore isolate (FJ445484) respectively. Four mutation aa residues in two Dongguan isolates were distributed in the nsP1 (T351A), nsP3 (P355L), E1 (S250P) and E2 (H313Y) regions respectively, but three mutation aa residues in two Yangjiang isolates were distributed in the nsP2 (T599I), nsP3 (S381Y) and C (A264V) regions respectively (Table 2). One aa residue located at the 226^th^ (1035 ^th^ site of polyprotein) site of the E1 gene was analyzed. A common aa change (A226V) was observed among four 2010 outbreak isolates (Table 2).

**Table 2:** Amino acid differences among China (2008, 2010), Singapore (2008) and Thailand (2009) isolates

**Gene**	**Polyprotein aa position**	**Specific protein aa position**	**SGEHICHT077808**	**CU-Chik683**	**FD080178**	**FD080008**	**GD113**	**GD115**	**GD134**	**GD139**
nsP1	351	351	Thr	Thr	Thr	Thr	Ala	Ala	Thr	Thr
nsP2	1134	599	Thr	Thr	Thr	Thr	Thr	Thr	Ile	Ile
nsP3	1688	355	Pro	Pro	Pro	Pro	Leu	Leu	Pro	Pro
	1714	381	Ser	Ser	Ser	Ser	Ser	Ser	Tyr	Tyr
C	264	264	Ala	Ala	Ala	Ala	Ala	Ala	Val	Val
E2	638	313	His	His	His	His	Tyr	Tyr	His	His
E1	1035	226	Val	Val	Val	Ala	Val	Val	Val	Val
	1059	250	Ser	Ser	Ser	Ser	Pro	Pro	Ser	Ser

## Discussion

CHIKV and dengue virus are both insect-borne viruses that can be propagated by *Ae*. *aegypti* and *Ae*. *albopictus*[27]. *Ae*. *albopictus* has a broad distribution in China, but *Ae*. *Aegypti* distributes mainly in the southern region of China, which includes Taiwan, the coast of Hainan, the western region of Guangdong Province, and the coast and several islands of Guangxi Province [28,29]. The Yangjiang and Dongguan regions of Guangdong Province have an abundant *Ae*. *albopictus* distribution, but *Ae*. *aegypti* is usually not found in these two regions [30]. Although CHIKV and dengue viruses share the same mosquito vector in Asia, outbreaks of the two viruses have not been frequently reported to occur in same region at the same time.

Coincidentally at a similar time to the CHIKV outbreaks, a dengue fever outbreak occurred in a suburb region of Dongguan, Guangdong Province in September 2010 [31]. Although, control measures were implemented during the dengue virus outbreak in this region, the vector was not effectively controlled within Dongguan city. Ineffective control measures increased the ability for CHIKV to rapidly spread in the same region in October leading to a CHIKV outbreak.

An average BI of 20 for *Ae*.*albopictus* was recorded from April to September in 2010 in Guangdong Province. The BI increased to around 40 after abundant raining during the end of September in Guangdong province, but a higher density of *Ae*. *albopictus* (BI=126) was observed during the CHIKV outbreak in the Huahong community. *Ae*. *aegypti* and *Ae*.*albopictus* are the main vector species known to transmit CHIKV in in Asia and the Indian Ocean region [27]. Therefore, we conclude that the particularly high densities of *Ae*. *albopictus* mosquitoes aided in promoting the CHIKV outbreak.

In Asia, the urban mosquito, *Ae*. *aegypti* has been found to be the most significant vector of CHIKV, with virtually all Asian mosquito isolates originating from this species [32]. Despite this, the pattern has been changed by an aa shift (A→V) at the 226^th^ position of the glycoprotein E1 in CHIKV. Both *Ae*. *aegypti* and *Ae*. *albopictus* are susceptible to the E1-A226V variant. However, the mutation caused enhanced replication and dissemination of the virus in *Ae*. *albopictus*, making it a more efficient vector of the variant [33]. This theory was proved correct by a large CHIKV outbreak that affected the Indian Ocean region from 2005–2006, with *Ae*. *albopictus* allowing an efficient replication and dissemination of CHIKV [15]. We hypothesize that mutation played a key role that resulted in two 2010 CHIKV outbreaks in the Guangdong province region. In order to verify the hypothesis, we analyzed the E1 genes of four virus stains isolated from the two outbreaks. The results revealed that the CHIKV causing the two outbreaks shared the same change of position of the A226V in the E1 gene. We infer that the E1-A226V variant of CHIKV aided CHIKV replication and dissemination by *Ae*.*albopictus* within the Guangdong region in 2010.

Three distinct CHIKV phylogenetic genotypes, the WAF genotype, the Asian genotype and the ECSA genotype, were identified in phylogenetic analysis based on previously identified complete genome sequences from around the world [23,34]. The Indian Ocean clade of the ECSA genotype was responsible for the largest outbreak that occurred in the Indian Ocean islands and India during 2005–2007 [15,18]. The previous study revealed that viruses spreading through the Indian Ocean originated in coastal Kenya during 2004, with closest known ancestors being members of the Central/East African clade [24].

To track the origin of the CHIKV that caused the two outbreaks in China, we collected genome representatives from three genotypes for phylogenetic analysis. The results showed the phylogenetic trees of CHIKV were similar with previous results. Four isolates from the two China outbreaks were located within the Indian Ocean clade of the ECSA genotype, but distributed in two different small branches, with the closest relationship to Singapore/SGEHICHT077808/2008 (FJ445484) and Thailand/CU-Chik683/2009 (GU301781) isolates. It was previously established that the 2008–2009 Singapore and Thailand outbreaks were caused by CHIKV E1-A226V strains imported from Kerala, India. However, these Indian isolates originally spread from Kenya independently [10,20]. Our findings indicate that strains from Singapore and Thailand did not disappear after 2009, but continued to persist, which caused the CHIKV introduction to China in 2010.

An outbreak of CHIKV was not reported in China until 2010. Interestingly, two outbreaks occurred successively in two regions during a two week intervals. We infer that the CHIKV causing the Dongguan outbreak was potentially carried into the Yangjiang region triggering a second outbreak. In order to clarify this hypothesis, the aa mutations were compared between the Dongguan and Yangjiang outbreak strains. Four common aa changes, distributed in the nsP1, nsP3, E1 and E2 regions respectively, were observed in two Dongguan strains, but were not found in the Yangjiang strains. As well, three common aa changes, distributed in the nsP2 and C regions respectively, were found in two Yangjiang strains and were not observed in the Dongguan strains. These results further help us conclude that the CHIKV that caused the two outbreaks might have originated from two different infectious sources. The conclusion is further supported by high bootstrap values for Figure 3.

## Conclusion

We described the outbreak of CHIKV that occurred in Yangjiang city of Guangdong Province in 2010, and identified the likely etiological agent to be CHIKV with an E1-A226V mutation. The high-density *Ae*. *albopictus* population was a contributing factor in the outbreak. We characterized the full genome of 4 CHIKV mutation strains from Yangjiang and Dongguang outbreaks. Sequence comparison, phylogenetic analyses, and evolutionary studies reveal that CHIKV throughout the world has been divided into 3 genotypes. The CHIKV causing the 2010 Guangdong outbreaks belonged to the Indian Ocean clade of the ECSA genotype and originated from the Southeast Asia isolates. This study also confirms variations of multiple aa between the two outbreak genomes, which helps us to infer that the CHIKV triggering the two 2010 outbreaks were from two different infectious sources.

## Material and methods

### Ethics statement

Use of sera, which was collected for this study, was approved by the Ethical Committee of the Centers for Disease Control and Prevention of Guangdong Province (GDCDC), and written informed consent was obtained from the study participants.

### Case definitions

For epidemiologic investigation purposes, GDCDC defines a clinical case of CHIK fever by sudden onset of fever (≥37.5°C) or arthralgia accompanied by maculopapular rashes or myalgia.

### CHIKV vector monitoring

*Aedes vigilax* density monitoring is performed monthly in monitoring sites according to the dengue fever and chikungunya fever surveillance programs in Guangdong Province. After the CHIKV cases were reported, investigation for *Ae*. *albopictus* habitats and BI were carried out every day in the outbreak community from Oct. 13 to Oct. 30, 2010. Staff from local CDCs implemented the site investigation in residents’ home and neighborhoods. Control measures, including cleaning out ponds to minimize breeding places, and spraying insecticides to kill mosquitoes, were simultaneously implemented.

### IgM and IgG detection

All of sera samples were tested for the presence of IgM or IgG antibodies against dengue and CHIKV. Dengue virus-specific antibodies were detected using a capture ELISA kit (Panbio., Brisbane, Australia) for IgM and for IgG according to the manufacturer’s instructions. CHIKV-specific antibodies were detected using an indirect immunofluorescence test (IIFT) (EUROIMMUN., Lübeck, Germany). In short, rheumatic factor was pre-adsorbed with EUROSORB reagent for the detection of IgM. The samples were diluted 1:10–1:80, and 25 μL were applied to the reaction fields of the BIOCHIPs, which were then incubated for 1 h. For antibody detection, anti-human IgG or IgM antibodies labeled with fluorescein isothiocyanate (FITC) were used. The results were evaluated by fluorescence microscopy; titers ≥1:10 were considered positive.

### RNA extraction

RNA was extracted from patient sera with a QIAamp Viral RNA Mini kit (QIAGEN Inc., Valencia, Calif). In short, 140 μl of each sample was treated with 560 μl of guanidine thiocyanate extraction buffer containing 10 μg/ml of carrier RNA, followed by alcohol precipitations. The precipitations were applied onto the QIAamp Mini columns, the viral nucleic acids were absorbed onto the silica-gel membrane. Finally, the pellet was resuspended in 50 μl of RNase-free water.

### LNA real-time RT-PCR probe examination

A LNA real-time RT-PCR amplification reaction was performed on 12 samples using the SuperScript TM III Platinum®OneStep Quantitative RT-PCR System with ROX (Invitrogen, USA). The reaction system consisted of 2×Reaction Mix with ROX (a buffer containing 0.4 mM of each dNTP, 6 mM MgSO4, and 1 μM ROX), 0.5 μl SuperScript™ III RT/ Platinum Taq Mix, and 0.8μl of 20 μM specific primers (CHIKVF: 5′-TTT AGC CGT AAT GAG CRT CGG-3′ / CHIKVR: 5′-CCG TGT TCG GGA TCA CTG TTA-3′). The CHIKV amplicons were detected with 1 μl of 10 μM LNA probe, labeled with FAM fluorophores at the 5′end and Black Hole Quencher (BHQ) at the 3′ end (FAM labelled: 5′-TGC *C*CA CA*C* TG*T* GA-3′ BHQ1, the italic nucleotides indicate a LNA monomer substitution), and 5 μl of extracted RNA was added to a final volume of 25 μl. The cycling conditions were: an initial cycle at 50°C for 15 min, and 94°C for 2 min; followed by 40 cycles at 95°C for 15 s, and 60°C for 30 s (data collection at stage 3, step 2). The Real-time RT-PCR reactions were performed in a BIO-RED C1000TM Thermal Cycler (Californai, USA).

### Virus isolation

The virus isolates in this study were isolated from the positive samples tested by Real-time RT-PCR. These samples were cultured in fresh monolayers of the BHK-21 and C6/36 cells. Cells were maintained in the medium supplemented with 10% fetal bovine serum (FBS). When the cells in monolayer presented 90% of confluence, the medium was discarded and 1 ml of diluted sample was added to a 24-well culture plate. Every sample was a 2-fold serial dilution from 1:50 to 1:1,600. Specimens were allowed to incubate at 33°C, 5% carbon dioxide and observed daily for cytopathic effect (CPE) for 7 days. Two blind passages were performed when no CPE was observed.

### Genome amplification by one-step RT-PCR

Fourteen-pair primers were designed according to CHKIV strain sequences (IND-KR52) from India. A one-step RT-PCR amplification reaction was performed by using the SuperScript TM III One-Step RT-PCR System with Platinum® Taq DNA Polymerase (Invitrogen, USA). The reaction system consisted of 2×Reaction Mix (a buffer containing 0.4 mM of each dNTP, 2.4 mM MgSO4), 0.5 μl SuperScript™ III RT/ Platinum Taq High Fidelity Enzyme Mix, 0.5 μM of the forward and reverse primers for other regions of genome and 5 μl of extracted RNA was added to a final volume of 25 μl. The cycling conditions for the ten RT-PCR genomes were: an initial cycle at 45°C 10 min, 50°C for 20 min and 94°C for 2 min; followed by 35 cycles at 94°C for 30 s, 45°C 30 s (increase in increments of 0.3°C for 1 second each up to 55°C) and 68°C for 1 min; and a final incubation at 68°C for 10 min. The 14 RT-PCR reactions were performed in Amplied Biosystems (Applied Biosystems, CA, USA). The band of PCR amplicons visualized after electrophoresis were subsequently excised from 1% agarose gel, and purified by use of a QIAGEN gel extraction kit (QIAGEN, Germany).

### Sequencing and genetic analysis

RT-PCR products were separated in a 1% agarose gel and stained with Gold View™ Nucleic Acid stain by electrophoresis. PCR products of the appropriate sizes were subsequently excised from the gel and purified by use of a QIAGEN gel extraction kit (QIAGEN, Germany). Nucleotide sequencing reactions were performed with a Bigdye terminator v3.1 cycle sequencing kit (Applied Biosystems) and resolved on an ABI 3100 Genetic Analyzer (Applied Biosystems). Sequencing reactions were subjected to the initial denaturation at 96°C for 2 min and 30 cycles consisting of 96°C for 10 sec, 50°C for 5 sec, and 60°C for 4 min in a Gene Amp PCR system 2700 (Applied Biosystems). The products were purified by use of the illustra Autoseq G-50 kit (Amersham Biosciences, UK).

To identify respective divergence and infer the genetic relationship among the isolates, the sequence analysis and comparisons were performed by using version 4.0 of the MEGA sequence analysis package. Phylogeny was analyzed and the resulting trees were constructed by using a neighbor-joining method of reconstruction phylogeny [35].

### Nucleotide sequence accession numbers

The genome sequences from this analysis are available in GenBank with the following accession numbers: HQ846356-HQ846359.

## Abbreviations

GDCDC: the Centers for Disease Control and Prevention of Guangdong Province; CHIKV: Chikungunya virus; BI: Breteau index; ELISA: Enzyme linked immunosorbent assay; IIFT: Indirect immunofluorescence test; WAf: the West African; ECSA: East/Central/South African; CPE: Cytopathic effect; FBS: Fetal bovine serum AA Amino acid; Nt: Nucleotide; LNA: Locked nucleic acid

## Competing interests

The authors declare that they have no competing interests.

## Authors’ contributions

WD carried out genetic analysis, drafted the manuscript. ZYH participated in the study design. ZHQ carried out viral isolation. KJ participated in RT-PCR test. LWJi and LWJa participated in sample collection. MC helped editing of the manuscript. ZH participated in indirect immunofluorescence test. ZHJ and ZQL performed field investigation. HJF performed field investigation. LH participated in whole-genome sequencing. SWC performed field investigation. KCW helped editing. LJY participated in the design of the study. All authors read and approved the final manuscript.

## References

[B1] Lahariya C Pradhan SK Emergence of chikungunya virus in Indian subcontinent after 32 years: a review J Vect Borne Dis 2006 43 4 151 160 17175699

[B2] Mason PJ Haddow AJ An epidemic of virus disease in Southern Province, Tanganyika Territory, in 1952–53; an additional note on Chikungunya virus isolations and serum antibodies Trans R Soc Trop Med Hyg 1957 51 3 238 240 10.1016/0035-9203(57)90022-6 13443013

[B3] Carey DE Chikungunya and dengue: a case of mistaken identity? J Hist Med Allied Sci 1971 26 243 262 4938938 10.1093/jhmas/xxvi.3.243

[B4] Paupy C Ollomo B Kamgang B Moutailler S Rousset D Demanou M Hervé JP Leroy E Simard F Comparative role of Aedes albopictus and Aedes aegypti in the emergence of Dengue and Chikungunya in central Africa Vector Borne Zoonotic Dis 2010 10 3 259 266 10.1089/vbz.2009.0005 19725769

[B5] Aikat BK Konar NR Banerjee G Hemorrhagic fever in Calcutta area Indian J. Med. Res 1964 52 660 675 14195506

[B6] Reuben R Some entomological and epidemiological observations on the 1964 outbreak of Chikungunya fever in South India Indian J Med Res 1967 55 1 1 12 6036047

[B7] Porter KR Tan R Suharyono Y Sutaryo W S W Ma'Roef C Listiyaningsih E Kosasih H Hueston L McArdle J Juffrie MA A serological study of Chikungunya virus transmission in Yogyakarta, Indonesia: evidence for the first outbreak since 1982 Southeast Asian J Trop Med Public Health 2004 2004 35 2 408 415 15691147

[B8] Thein S La Linn M Aaskov J Aung MM Aye M Zaw A Myint A Development of a simple indirect enzyme-linked immunosorbent assay for the detection of immunoglobulin M antibody in serum from patients following an outbreak of chikungunya virus infection in Yangon, Myanmar Trans R Soc Trop Med Hyg 1992 86 4 438 442 10.1016/0035-9203(92)90260-J 1332222

[B9] Noridah O Paranthaman V Nayar SK Masliza M Ranjit K Norizah I Chem YK Mustafa B Kumarasamy V Chua KB Outbreak of chikungunya due to virus of Central/East African genotype in Malaysia Med J Malaysia 2007 62 4 323 328 18551938

[B10] Leo YS Chow AL Tan LK Lye DC Lin L Ng LC Chikungunya outbreak, Singapore, 2008 Emerg Infect Dis 2009 15 5 836 837 10.3201/eid1505.081390 19402989 PMC2687008

[B11] Thavara U Tawatsin A Pengsakul T Bhakdeenuan P Chanama S Anantapreecha S Molito C Chompoosri J Thammapalo S Sawanpanyalert P Siriyasatien P Outbreak of chikungunya fever in Thailand and virus detection in field population of vector mosquitoes, Aedes aegypti (L.) and Aedes albopictus Skuse (Diptera: Culicidae) Southeast Asian J Trop Med Public Health 2009 40 5 951 962 19842379

[B12] Chastel C Human Infections in Cambodia By the Chikungunya Virus or An Apparently Closely Related Agent. II. Experimental Pathological Anatomy Bull Soc Pathol Exot Filiales 1963 56 915 924 14127358

[B13] Vu QD Nguyen TKT Ly QB Study of anti-Chikungunya antibodies in Vietnamese children in Saigon Bull Soc Pathol Exot Filiales 1967 60 14 353 359 5632304

[B14] Bordi L Carletti F Castilletti C Chiappini R Sambri V Cavrini F Ippolito G Di Caro A Capobianchi MR Presence of the A226V mutation in autochthonous and imported Italian chikungunya virus strains Clin Infect Dis 2008 47 428 429 10.1086/589925 18605910

[B15] Vazeille M Moutailler S Coudrier D Rousseaux C Khun H Two Chikungunya Isolates from the Outbreak of La Reunion (Indian Ocean) Exhibit Different Patterns of Infection in the Mosquito Aedes albopictus. PLoS ONE 2007 2 1168 10.1371/journal.pone.0001168 PMC2064959 18000540

[B16] Borgherini G Poubeau P Staikowsky F Lory M Le MN Becquart JP Wengling C Michault A Paganin F Outbreak of chikungunya on Re´union Island: early clinical and laboratory features in 157 adult patients Clin Infect Dis 2007 44 1401 1407 10.1086/517537 17479933

[B17] Kumar NP Suresh A Vanamail P Sabesan S Krishnamoorthy KG Mathew J Jose VT Jambulingam P Chikungunya virus outbreak in Kerala, India, 2007: a seroprevalence study Mem Inst Oswaldo Cruz 2011 106 8 912 916 22241110 10.1590/s0074-02762011000800003

[B18] Chretien JP Anyamba A Bedno SA Breiman RF Sang R Sergon K Powers AM Onyango CO Small J and other authors Drought-associated chikungunya emergence along coastal East Africa. AmJTrop Med Hyg 2007 76 405 407 17360859

[B19] Srikanth P Sarangan G Mallilankaraman K Nayar SA Barani R Mattew T Selvaraj GF Sheriff KA Palani G Muthumani K Molecular characterization of Chikungunya virus during an outbreak in South India Indian J Med Microbiol 2010 28 4 299 302 10.4103/0255-0857.71812 20966558

[B20] Rianthavorn P Prianantathavorn K Wuttirattanakowit N Theamboonlers A Poovorawan Y An outbreak of chikungunya in southern Thailand from 2008 to 2009 caused by African strains with A226V mutation Int J Infect Dis 2010, 4 Suppl 2010 4 161 165 10.1016/j.ijid.2010.01.001 20417142

[B21] Apandi Y Lau SK Izmawati N Amal NM Faudzi Y Mansor W Hani MH Zainah S Identification of Chikungunya virus strains circulating in Kelantan, Malaysia in 2009 Southeast Asian J Trop Med Public Health 2010 41 6 1374 1380 21329313

[B22] Enserink M Entomology. A mosquito goes global Science 2008 320 864 866 10.1126/science.320.5878.864 18487167

[B23] Shu PY Yang CF Su CL Chen CY Chang SF Tsai KH Cheng CH Huang JH Two imported chikungunya cases Taiwan. Emerg Infect Dis 2008 14 8 1326 1327 10.3201/eid1408.071304 PMC2600404 18680674

[B24] Zheng K Li J Zhang Q Liang M Li C Lin M Huang J Li H Xiang D Wang N Hong Y Huang L Li X Pan D Song W Dai J Guo B Li D Genetic analysis of chikungunya viruses imported to mainland China in 2008 Virol J 2010 18 7 8 20078896 10.1186/1743-422X-7-8 PMC2831882

[B25] Wu D Wu J Zhang Q Zhong H Ke C Deng X Guan D Li H Zhang Y Zhou H He J Li L Yang X Emerg Infect Dis 2012 Mar 18 3 493 495 10.3201/eid1803.110034 22377135 PMC3309566

[B26] Kariuki Njenga M Nderitu L Ledermann JP Ndirangu A Logue CH Kelly CH Sang R Sergon K Breiman R Powers AM Tracking epidemic Chikungunya virus into the Indian Ocean from East Africa J Gen Virol 2008 89 Pt 11 2754 2760 18931072 10.1099/vir.0.2008/005413-0 PMC3347796

[B27] Pialoux G Gaüzère BA Jauréguiberry S Strobel M Chikungunya, an epidemic arbovirosis Lancet Infect Dis 2007 7 5 319 327 10.1016/S1473-3099(07)70107-X 17448935

[B28] Xie H Zhou HN Yang YM Advances in the research on the primary dengue vector *Aedes aegypti* in China Chin J Vector Biol & Control 2011 22 2 194 19716030375

[B29] Gong DF Zhou HN Progress in Dengue fever important vector *Aedes albopctus* in China Chin J Vector Biol & Control 2009 20 6 607 61016030375

[B30] Zhou HK Tang XY Huang Q Distribution of Aedes aegypti in China and its comprehensive control Zhonghua Liu Xing Bing Xue Za Zhi 1982 3 6 354 356 7185486

[B31] Peng HJ Lai HB Zhang QL Xu BY Zhang H Liu WH Zhao W Zhou YP Zhong XG Jiang S Duan JH Yan GY He JF Chen XG A local outbreak of dengue caused by an imported case in Dongguan China BMC Publ Health 2012 26 12 83 10.1186/1471-2458-12-83 PMC3311058 22276682

[B32] Thiboutot MM Kannan S Kawalekar OU Shedlock DJ Khan AS Sarangan G Srikanth P Weiner DB Muthumani K Chikungunya: a potentially emerging epidemic? PLoS Negl Trop Dis 2010 4 4 e623 10.1371/journal.pntd.0000623 20436958 PMC2860491

[B33] Tsetsarkin KA Vanlandingham DL McGee CE Higgs S A single mutation in chikungunya virus affects vector specificity and epidemic potential PLoS Pathog 2007 3 12 201 10.1371/journal.ppat.0030201 PMC2134949 18069894

[B34] Volk SM Chen R Tsetsarkin KA Adams AP Garcia TI Sall AA Nasar F Schuh AJ Holmes EC Higgs S Maharaj PD Brault AC Weaver SC Genome-scale phylogenetic analyses of chikungunya virus reveal independent emergences of recent epidemics and various evolutionary rates J Virol 2010 84 13 6497 6504 10.1128/JVI.01603-09 20410280 PMC2903258

[B35] Tamura K Dudley J Nei M Kumar S MEGA4: Molecular Evolutionary Genetics Analysis (MEGA) software version 4.0 Mol Biol Evol 2007 24 1596 1599 10.1093/molbev/msm092 17488738

